# MET Expression Level in Lung Adenocarcinoma Loosely Correlates with *MET* Copy Number Gain/Amplification and Is a Poor Predictor of Patient Outcome

**DOI:** 10.3390/cancers14102433

**Published:** 2022-05-14

**Authors:** Wei Yin, Ming Guo, Zhenya Tang, Gokce A. Toruner, Joanne Cheng, L. Jeffrey Medeiros, Guilin Tang

**Affiliations:** 1 Department of Hematopathology, The University of Texas MD Anderson Cancer Center, Houston, TX 77030, USA; yinwei2177@csu.edu.cn (W.Y.); ztang@mdanderson.org (Z.T.); gatoruner@mdanderson.org (G.A.T.); jtcheng@mdanderson.org (J.C.); ljmedeiros@mdanderson.org (L.J.M.); 2 Department of Thoracic Surgery, The Second Xiangya Hospital of Central South University, Changsha 410011, China; 3 Department of Pathology, The University of Texas MD Anderson Cancer Center, Houston, TX 77030, USA; mguo@mdanderson.org

**Keywords:** MET expression, *MET* amplification, lung cancer, survival

## Abstract

**Simple Summary:**

*MET* is a proto-oncogene and plays an important role on tumor cell survival, proliferation, metastasis, and drug resistance. Patient with *MET* amplification has shown an inferior outcome comparing to patients without *MET* amplification. Fluorescence in situ hybridization (FISH) is often used to detect *MET* amplification, and immunohistochemistry (IHC) is often used to assess MET expression level. Though some institutions provide both tests, IHC is more readily available in most pathology laboratories and is cheaper than FISH. This study evaluated the correlation of MET expression level with *MET* copy number gain/amplification, and the MET overexpression with patient’s outcome. By studying 446 patients with lung adenocarcinoma, we found that the concordance of MET expression and *MET* copy number gain/amplification was low; high-level of MET expression was associated with inferior outcome, but it was not an independent poor prognostic factor. These findings indicate that IHC for MET expression can’t substitute FISH analysis for *MET* amplification.

**Abstract:**

*MET* amplification has been associated with shorter survival in cancer patients, however, the potential correlation of MET overexpression with either *MET* amplification or patient outcome is controversial. The aim of this study was to address these questions by correlating MET expression level with *MET* copy number and patient outcome in a cohort of 446 patients who had a lung adenocarcinoma: 88 with *MET* amplification, 118 with polysomy 7, and 240 with negative results by fluorescence in situ hybridization. MET expression assessed by immunohistochemistry was semi-quantified by expression level: absent (0+), weak (1+), moderate (2+) and strong (3+); or by H-score: 0–99, 100–199, and ≥200. MET expression level or H-score was positively but weakly correlated with *MET* copy number or *MET/CEP7* ratio. Strong expression of MET (3+ or H-score ≥ 200) was associated with a shorter overall survival, but it was not an independent hazard for survival by multivariant analysis. We conclude that MET expression is loosely correlated with MET copy number gain/amplification. Strong expression of MET does not independently predict patient outcome.

## 1. Introduction

*MET*, located on chromosome 7q31, is an oncogenic receptor tyrosine kinase for hepatocyte growth factor. Activation of MET pathway, either by *MET* amplification or a splice site alteration in exon 14, plays important roles on tumor cell survival, proliferation, metastasis, and drug resistance [1,2,3]. Evidence from preclinical and clinical trials suggests MET activation serves as a primary oncogenic driver in a subset of patients with non-small cell lung cancer (NSCLC) and as a secondary driver of acquired resistance to targeted therapy in *EGFR*-mutant [3] or *ALK*-positive patients [4]. Recent investigations have shown that a subset of patients harboring *MET* exon 14 mutation or *MET* amplification can benefit from MET inhibitors [5,6,7].

Fluorescence in situ hybridization (FISH) has been commonly used to detect *MET* copy number change or *MET* amplification. *MET* amplification often exhibits a high copy number of *MET* (≥15 copies or clusters of *MET* signals) and/or a *MET/CEP7* (centromere of chromosome 7) ratio ≥ 1.8 [8,9] or ≥2.0 [10,11] by FISH. *MET* amplification has been detected in 1% to 5.8% of treatment-naïve NSCLC patients [9,10,11,12,13,14,15,16,17] and in 5% to 26% of patients with *EGFR*-mutant NSCLC who develop resistant to EGFR inhibitors [13,14,15,16,18,19,20]. *MET* amplification has been associated with an inferior outcome [9,11,12,21,22,23,24]. Immunohistochemistry (IHC) is a widely used assay to assess gene expression level, it is more readily available than FISH in most pathology laboratories and is more cost-effective than FISH. The frequency of MET overexpression in unselected NSCLCs ranges from 15% to 70%, mainly depending on the criteria used to “define” overexpression [17,24,25,26,27,28,29,30,31]. Some studies have suggested that MET express level can serve as a potential predictive marker for overall survival (OS) and/or progression-free survival, and the likelihood that patient responds to MET kinase inhibitor [24,30,31,32,33]. On the contrary, other studies have not found an association between MET expression level and patient survival [25,27,28,29]. Others have suggested that MET IHC is not a good screening test for *MET* amplification or *MET* exon 14 mutation in lung cancer [17,26]. Previous clinical trials that focused on MET pathway–directed targeted therapy in unselected or MET-overexpressing NSCLC patients have yielded largely negative results [3].

In this retrospective study, we included 446 patients with lung adenocarcinoma, we evaluated the correlations between *MET* copy number gain /amplification (by FISH) and MET expression level (by IHC). We also evaluated whether overexpression of MET can predict patient outcome.

## 2. Materials and Methods

### 2.1. Study Cohort

The study group included patients who were diagnosed with lung adenocarcinoma at our institution during a six-year period (January 2014 to December 2019), and who had been evaluated for *MET* copy number/amplification by FISH and MET expression by IHC. Patient follow-up and outcomes were obtained by electronic chart review. This study was approved by the Institutional Review Board at The University of Texas MD Anderson Cancer Center and was conducted in accord with the Declaration of Helsinki.

### 2.2. FISH Analysis

**Figure 1:**
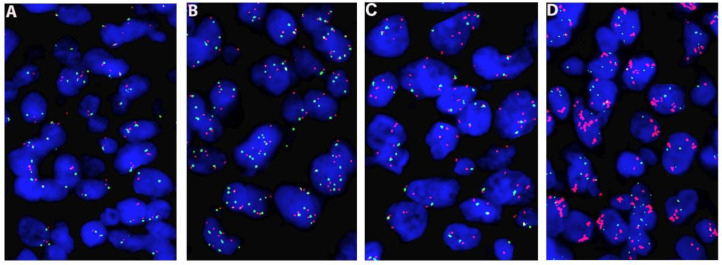
Fluorescence in situ hybridization (FISH) analysis using *MET/CEP7* probes (×60). (**A**): MET negative; (**B**): Polysomy 7 (copy number gain detected in both *MET* and *CEP7*); (**C**): *MET* amplification (copy number gain in *MET*, not in *CEP7*, *MET/CEP7* ratio > 1.8); (**D**): *MET* amplification (clusters of *MET* signals). FISH probe signals: *MET* in red and centromere 7 (*CEP7*) in green.

FISH analysis for *MET*/*CEP7* (*MET* labeled as red and *CEP7* labelled as green, Biocare Medical, LLC., Concord, CA) was performed on formalin-fixed paraffin-embedded (FFPE) tissue sections as a part of clinical workup for all newly diagnosed NSCLC at our institution as described previously [9]. After hybridization and post-wash, DAPI was applied to each slide. Fifteen fields or whole tissue section were scanned by Metafer automated imaging system (MetaSystems). Technologists analyzed sixty tumor cells (with relatively large nuclei) by counting signals of both *MET* and *CEP7* and calculated the *MET/CEP7* ratio for each specimen. For cells with clusters of *MET* signals, the copy number of *MET* was recorded as 20 for calculation purpose. *MET* amplification (MET-amp) was considered when one or more of these criteria were met: the *MET/CEP7* ratio was ≥1.8; >10% of cells showed clusters of *MET* signals; or MET copy ≥ 15. Polysomy 7 was considered if *MET/CEP7* ratio < 1.8 and *MET* copy ≥ 5 but <15. The remaining cases were designated as *MET* negative (MET-neg) [9] (Figure 1).

### 2.3. Immunohistochemistry Stain

**Figure 2:**
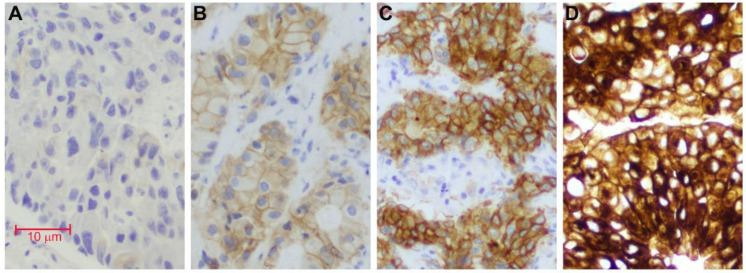
MET expression level by immunohistochemistry stain (×20). (**A**): Absent (0+); (**B**): Weak (1+); (**C**): Moderate (2+); (**D**): Strong (3+).

MET IHC was performed on FFPE tissue sections using anti-total MET rabbit monoclonal antibody (clone SP44, Roche Diagnostics, dilution 1:1). MET expression intensity (level) and percentage of cells were evaluated and recorded by two pathologists independently. In tumors that exhibited intra-tumor heterogeneous staining intensity, the predominant (≥50% of tumor cells) staining pattern was recorded. The intensity was classified as absent (IHC0+), weak (IHC1+), moderate (IHC2+) and strong (IHC3+) (Figure 2, ×20). In addition to the staining intensity, H-score was also assessed. The H-score was calculated by a formula including both intensity and percentage of cells: (3× percentage of cells with IHC3+) + (2× percentage of cells with IHC2+) + (1× percentage of cells with IHC1+). H-score ranged from 0 to 300.

### 2.4. Statistical Analysis

Spearman correlation analysis was used to analyze the correlation coefficient (r) between MET expression level (IHC or H-score) and *MET* copy number (or *MET/CEP7* ratio). Chi-square was used to compare categorical variables. Overall survival (OS) was estimated by Kaplan-Meier method from the date of diagnosis of lung adenocarcinoma to the date of death from any cause or censored at time of last follow-up for living patients. Multivariate cox proportional hazard regression analysis was used to assess the relationship between survival and age, tumor stage, *MET* FISH groups and MET overexpression (IHC3+ or H-score ≥ 200). The difference was considered as significant when *p* < 0.05.

## 3. Results

### 3.1. Patients

During this study period, there were 1987 patients who had FISH for *MET/CEP7* tested on the first diagnostic specimen of lung adenocarcinoma in our institution, including 112 (5.6%) patients with *MET/CEP* ratio ≥ 1.8 (MET-amp), 146 (7.3%) with polysomy 7, and 1729 (87%) with a MET-neg FISH results.^9^ Among the 258 patients with MET-amp or polysomy 7, 206 (88 with MET-amp and 118 with polysomy 7) had materials available for IHC. In addition, we included a comparable number (n = 240) of patients from MET-neg group who had IHC available and who showed similar distribution of age, gender, tumor stage, and diagnostic year compared to the groups of MET-amp and polysomy 7.

Among the 446 patients included in this study cohort, 95 (21.3%) patients with stage I/II and 351 (78.7%) patients with stage III/IV disease. The sex and age distribution are shown in Table 1.

### 3.2. Immunohistochemistry

MET IHC exhibits membranous and cytoplasmic reactivity, and the IHC pattern was relatively homogenous in most cases. The expression level was determined on the basis of the stain intensity and fraction of positive cells by two pathologists. In most cases (n = 392, 88%) observations by both pathologists were concordant. Discordance occurred in 54 patients: 18 with 1+ or 2+ and 36 with 2+ or 3+. The discordant cases were reviewed by the third pathologist. After consensus, 41 (9.2%) neoplasms were negative for MET (0+), 55 (12.3%) were weakly positive (1+), 231 (51.8%) were moderately positive (2+), and 119 (26.7%) were strongly positive (3+). H-score fell into 0–99 in 96 (21.5%) cases; 100–199 in 205 (46%) and ≥200 in 145 (32.5%) patients (Table 2).

### 3.3. Correlation of MET Expression with MET Copy Number or MET/CEP7 Ratio

**Figure 3:**
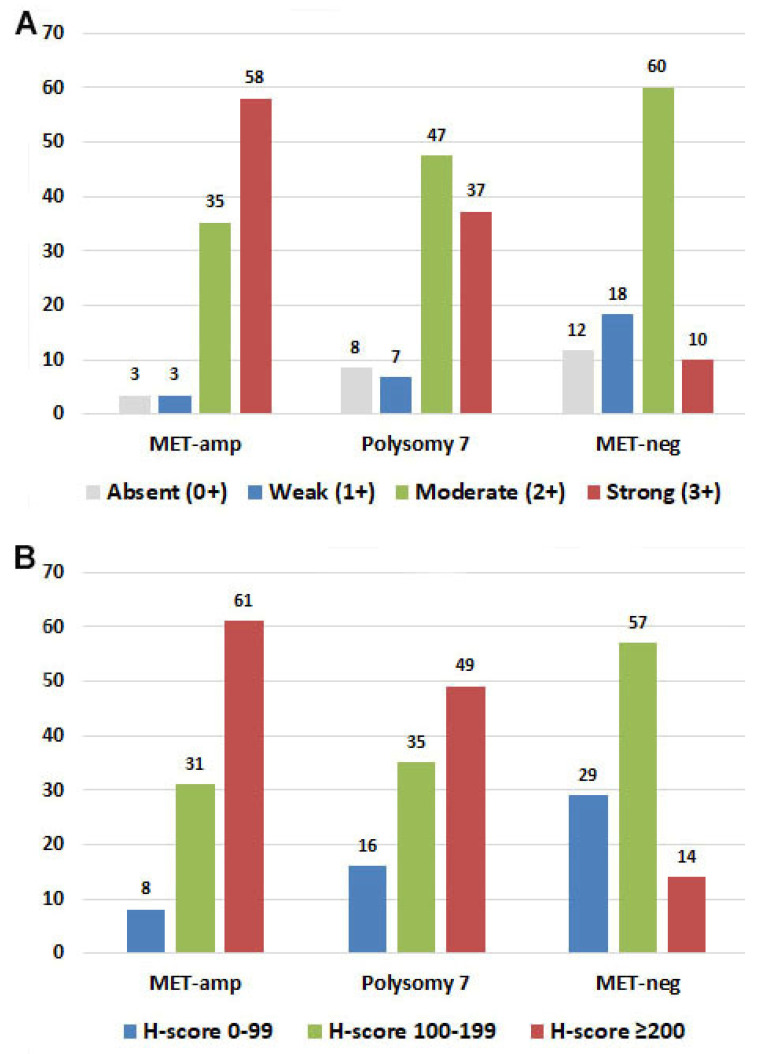
Correlation of FISH groups and immunohistochemistry results. (**A**): Proportion of patients with different level of MET expression (IHC0+~3+) in patients with MET-amp, Polysomy 7 and MET-neg. (**B**): Proportion of patients with MET H-score of 0–99, 100–199 and ≥200 in patients with MET amp, Polysomy 7 and MET neg. Presented as percentage of cases.

MET expression level (IHC 0–3) and MET H-score were loosely and positively correlated with *MET* copy number (*p* < 0.0001) or *MET/CEP7* ratio (*p* < 0.0001), with a correlation coefficient (r) ranged from 0.4298 to 0.4431 (Table 2). MET expression level (or H-score) was significantly different (*p* < 0.0001) among patients with MET-amp, polysomy 7 or MET-neg (Table 2, Figure 3). High proportion (58%) of cases in the MET-amp group showed strong (3+) MET expression versus 37% in the polysomy 7 group and 10% in the MET-neg group. Similarly, high proportion (61%) of cases in the MET-amp group showed H-score ≥ 200 comparing to 49% in polysomy 7 group and 14% in MET-neg group. Patients with MET-neg and polysomy 7 more commonly showed moderate (IHC2+) expression of MET, 60% and 47%, respectively.

### 3.4. Correlation of MET Expression and Tumor Stage

MET expression level and tumor stage are summarized in Table 1; strong (IHC3+) expression was associated with advanced (III/IV) tumor stage (*p* < 0.0001). Similarly, H-score ≥ 200 was also associated with advanced tumor stage (*p* = 0.0021).

### 3.5. Correlation of MET Expression and Patient Outcome

**Figure 4:**
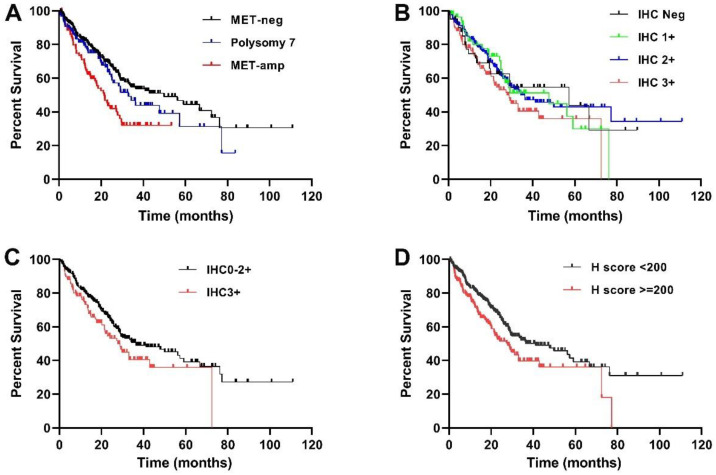
Overall survival (OS) by Kaplan-Meier analysis. (**A**): Comparison of OS among patients with MET-amp, polysomy 7 and MET-neg, patients in MET-amp group showed significantly inferior OS; (**B**): No significant difference of OS among patients of groups with of IHC-neg, IHC1+, IGHC2+ and IHC3+; (**C**): Patients with IHC3+ showed a significantly shorter OS comparing to patients with IHC0/1+/2+; (**D**): Patients with H- score ≥ 200 showed a significantly inferior OS comparing to patients with H score < 200.

As reported previously [9], MET-amp was significantly associated with inferior survival. The median overall survival of three FISH groups (MET-amp, polysomy 7 and MET-neg) was 21.4 months, 33 months and 49.8 months, respectively (*p* = 0.0005, Figure 4A). The clinical outcomes of patients in the MET IHC groups are summarized in Table 1. Median overall survival was 57.2 months for patients with tumors showing no MET expression, versus 47.7 months, 34.3 months, 28.8 months for patients with weak (1+), moderate (2+), and strong (3+) MET expression, respectively (*p* = 0.0966). Although these data show a trend that patients with a higher level of MET expression (1+~3+) had a shorter survival comparing to the patients without MET expression (0+), but the differences were not significant (*p* = 0.2694, 0.6625, and 0.1927 for IHC 1+, 2+, 3+ comparing to IHC 0+) (Figure 4B).

We then analyzed whether a higher MET expression (IHC2+ or IHC3+) could predict patient’s survival. As shown in Table 3, patients with IHC3+ showed a significant shorter median OS comparing to the patients with IHC0/1+/2+ (28.8 vs. 36.3 months, *p* = 0.0463, Figure 4C); however, the significance was not seen among patients with IHC0/1+ and patients with IHC2+/3+ (*p* = 0.6630) (Table 3).

We also analyzed whether a high H-score (≥150 or ≥200) could predict patient’s outcome. As shown in Table 3, patients with H-score ≥ 200 showed a significant shorted median OS (27.8 vs. 41.1 months, *p* = 0.0110, Figure 4D) comparing to patients with H-score < 200, but patients with H-score ≥ 150 and patients with H-score < 150 showed comparable OS (*p* = 0.2950) (Table 3).

Though MET-amp, IHC3+ and H-score ≥ 200 were significantly associated with inferior survival in the univariate analysis, only MET-amp remained (in addition to older age and advanced stage) to be prognostically significant by multivariate analysis when *MET* FISH, age (≥65 years), tumor stage (III/IV), and MET expression (either with ^a^ IHC3+ or with ^b^ H-score ≥ 200) were co-analyzed for OS (Table 4). When age, tumor stage, and MET expression were included (without MET FISH), only older age and advanced tumor stage were significant hazards to survival (*p* < 0.0001). High-level MET expression (either IHC3+ or H-score ≥ 200) was not a significant hazard to survival.

## 4. Discussion

*MET* is an oncogenic receptor tyrosine kinase. In vitro and in mice, overexpression of MET alone is sufficient to induce oncogenic transformation [34,35]. Many studies have explored the potential of MET overexpression, as assessed by IHC, to serve as a prognostic marker and/or predictive marker for *MET* amplification, *MET* exon 14 mutation, or the response to MET kinase inhibitors. To date, the results of these studies have been contradictory (Table 5). Currently, no consensus on the definition of “positive” MET expression has been reached. Some studies used stain intensity of 2+ [27,28,29] or 3+ [36] (scale of 0+ to 3+); some studies used H-score (0–300), ≥20 [30], ≥150 [26], or ≥200 [17]; some studies multiplied the intensity score and the fraction score (range 0-12) and consider positive when score fell into 4–12 [24]. In large part related to this lack of consensus, the rate of MET expression has differed greatly in these studies, from 15% to 70% [17,24,25,26,27,28,29,30,31]. (Table 5). In the current study, we used IHC3+ or H-score ≥ 200 as cut-off, both predicted an inferior survival in the univariate analysis, but not in multivariate analysis.

The design of this study is slightly different from the studies published previously. First, this study cohort included a large number of patients with MET-amp (n = 88) and polysomy 7 (n = 118), in contrast with most published studies in which a smaller number of patients with MET-amp and polysomy 7 were included (due to the low frequency of MET-amp or polysomy 7 in treatment-naïve NSCLC in general). This higher proportion of cases with MET-amp and polysomy 7 enabled us to do a better comparison of MET expression and MET copy number gain/amplification. Second, we analyzed the association of high-level expression of MET (IHC3+ or H-score ≥ 200) with survival by both univariate and multivariate analysis, though high-level of MET expression was associated with inferior survival by univariate analysis, it was not a significant hazard to survival by multivariate analysis when age and tumor stage were included in analysis. Third, we also analyzed the association of spectrum of stain intensities (0+~3+) with FISH results (*MET* copy number and *MET/CEP7* ratio) and patient outcome.

We compared the association of *MET* copy number or *MET/CEP7* ratio by FISH and MET expression by intensity or H-score in parallel, to explore whether IHC could possibly replace FISH. The results of this study showed a weakly positive correlation of MET expression with *MET* copy number or *MET/CEP7* ratio (r ranged 0.4298 to 0.4431, *p* < 0.0001), which was in line with other studies [24,26,27,28,29]. In patients with MET-amp, ~58% of patients showed strong (3+) MET expression. However, IHC3+ was also seen in the polysomy 7 (~37%) and MET-neg (~10%) groups. Similar findings were also observed in H-score. The sensitivity of using IHC3+ was 43% and the specificity was 89% for predicting MET-amp in this cohort, which is similar to an earlier study by Mignard and colleagues [26]. Among the patients with polysomy 7 and MET-neg FISH results, IHC 2+ was the most common result, 47% in the polysomy 7 and 60% in MET-neg groups, respectively.

Multiple factors could contribute to the low concordance rate between *MET* expression and MET gene copy number (GCN). Biologically, MET expression is not only controlled by *MET* GCN, but also by transcriptional and/or translational regulation. Besides, intratumoral heterogeneity [15,37], sampling (small core biopsy or tissue microarray), tumor histology type and differentiation, and tumor load at a metastatic site also could contribute to a low concordance between MET expression and MET GCN. Among the cases with a strong MET expression (IHC3+ or H-score ≥ 200) but a negative FISH result, a common feature we have observed was low tumor infiltrations in the metastatic tissue biopsy specimens. We also noticed that tumors with MET-amp, but with 0+/1+ MET expression tended to be poorly differentiated.

*MET* amplification, IHC3+ or H-score ≥ 200 was significantly associated with inferior survival by univariate analysis, however, IHC3+ or H-score ≥ 200 was not an independent hazard to survival by multivariate analysis when age and stage were included (with or without *MET* FISH groups). This could be due to the fact that high expression of MET (IHC3+ or H-score ≥ 200) was highly associated with advanced tumor stage [33]. Thus, our findings support the interpretation that MET expression level is a poor predictor of patient survival [25,27,28,29].

In summary, the concordance between MET expression and *MET* GCN/amplification is low. Unlike *MET* amplification highly associates with a short survival, MET expression level is not significant hazard to survival. MET expression level by IHC can’t substitute FISH analysis for MET copy gain/amplification.

## 5. Conclusions

Apart from previous studies, this study cohort included much higher proportion of cases showing MET-amp (88, ~20%) and polysomy 7 (118, ~26%), in addition to 240 (54%) cases with MET-neg. We found that MET expression was weakly and positively associated with *MET* copy or *MET/CEP7* ratio. High-level MET expression (IHC3+ or H-score ≥ 200), though seemed to be associated with an inferior survival, was not an independent hazard to survival.

## Figures and Tables

**Table 1:** Associations of MET expression level with *MET* copy number, cancer stage and outcome.

	Absent (0+) (n = 41)	Weak (1+) (n = 55)	Moderate (2+) (n = 231)	Strong (3+) (n = 119)	Total (n = 446)
Age (range)	68 (33–85)	68 (44–83)	67 (29–94)	65 (29–83)	66 (29–94)
Gender (M/F)	22/19	30/25	100/131	51/68	203/243
**FISH analysis**					
MET-amp	3 (3.4%)	3 (3.4%)	31 (35.2%)	51 (58%)	88 (19.7%)
Polysomy 7	10 (8.5%)	8 (6.8%)	56 (47.5%)	44 (37.3%)	118 (26.5%)
MET-neg	28 (11.7%)	44 (18.3%)	144 (60%)	24 (10%)	240 (53.8%)
**Cancer stage**					
I/II	15 (36.6%)	12 (21.8%)	59 (25.5%)	9 (7.6%)	95 (21.3)
III/IV	26 (66%)	43 (78.2%)	172 (74.5%)	110 (92.3%)	351 (78.7)
**Outcome**					
Alive/Dead	23/18	30/25	139/92	63/56	255/191
Median OS (mon)	57.2	47.7	34.3	28.8	

FISH: fluorescence in situ hybridization; M/F: male/female; mon: months; neg: negative; OS: overall survival.

**Table 2:** Correlation of MET FISH groups with MET expression (IHC level or H-score).

	MET-amp (n = 88)	Polysomy 7 (n = 118)	MET-neg (n = 240)	Total (n = 446)	Correlation (r)	*p*
IHC0	3 (7%)	10 (24%)	28 (68%)	41	^a^ 0.4431 ^b^ 0.4401	<0.0001 <0.0001
IHC1+	3 (5%)	8 (15%)	44 (80%)	55		
IHC2+	31 (13%)	56 (24%)	144 (62%)	231		
IHC3+	51 (43%)	44 (37%)	24 (20%)	119		
H-score 0–99	7 (7%)	19 (20%)	70 (73%)	96	^a^ 0.4381 ^b^ 0.4298	<0.0001 <0.0001
H-score 100–199	27 (13%)	41 (20%)	137 (67%)	205		
H-score 200–00	54 (37%)	58 (40%)	33 (23%)	145		

Correlation coefficient of MET expression with ^a^ MET copy number or ^b^ MET/CEP7 ratio.

**Table 3:** Association of MET expression with survival by using IHC2+/IHC3+ or H-score ≥ 150 or ≥200 as cut-off (Kaplan-Meier method).

	No. of Patients	Median Survival	*p*
IHC0/1+	96	47.7	0.6630
IHC2+/3+	350	33	
IHC0/1+/-2+	327	36.3	0.0463
IHC3+	119	28.8	
H-score < 150	122	47.5	0.2950
H-score ≥ 150	324	32.8	
H-score < 200	301	41.1	0.0110
H-score ≥ 200	145	27.8	

**Table 4:** Multivariate cox proportional hazard regression analysis for overall survival.

	^a^ Overall Survival		^b^ Overall Survival	
Variables	Hazard Ratio (95% CI)	*p*	Hazard Ratio (95% CI)	*p*
**Age** (≥65 vs. <65 years)	1.849 (1.372–2.491)	<0.0001	1.827 (1.355–2.462)	<0.0001
**Stage** (I/II vs. III/IV)	4.163 (2.560–6.770)	<0.0001	4.215 (2.585–6.873)	<0.0001
***MET* FISH**				
Polysomy 7 vs. MET-neg	1.046 (0.729–1.501)	0.588	1.100 (0.769–1.573)	0.602
MET-amp vs. MET-neg	1.407 (1.057–2.311)	0.045	1.547 (1.067–2.422)	0.027
**Immunohistochemistry**				
**H-score** (≥200 vs. <200)	1.200 (0.865–1.664)	0.275		
**IHC** (IHC0-2+ vs. IHC3+)			0.984 (0.694–1.396)	0.930

These were two analyses: H-score (left, a) or IHC (right, b) was included in separate analysis, while age, stage and *MET* FISH were included in both analyses.

**Table 5:** Current and Previous Studies on MET Overexpression and the Associations with *MET* Copy Number /Patient Survival.

Reference	Criteria for MET Overexpression	Positive Rate	Tissue	Antibody	Correlates with *MET* GCN?	Associates with Survival?
Bubendorf (2017) [28]	>50% exhibit 2+ staining	23.8%	TMA	SP44	Yes	Not correlated
Dziadziuszko (2012) [29]	METMab: ≥50% of cells with ≥2+	25% (44/174)	TMA	SP44	Yes	Not correlated
Guo (2019) [17]	H-score ≥ 200	39% (71/181)		SP44	Poor	NA
Mignard (2018) [26]	H-score ≥ 150 MetMab Score: 2+/3+	15/81(18.5%) 14/81 (17%)		SP44	Poor	NA
	MetMab Score: 2+/3+					
Park (2012) [24]	4–12 (of 0–12 scale) (intensity x fraction)	13.7 (52/380)		3D4 (Rabbit polyclonal	Yes	Significant shorter OS and DFS
Rivalland (2019) [27]	>50% exhibit 2+ staining	25% (193/763)	TMA	SP44	Yes	Not correlated
Tsuta (2012) [25]	Cytoplasmic/membrane, ≥10% cells	22.2%		SP44	NA	Not correlated
Weingertner (2015) [31]	METMab: ≥50% of cells ≥2+	44% (89/201)	TMA	SP44	High GCN often have overexpression; ~1/3 overexpression cases had high *MET* GCN	Only in non-smoke group (32/201)
	3+ in ≥10% of cells	28% (57/201)				
	H-score: >140	42% (84/201)				
**Current study**	IHC3+, ≥50% of cells	26.7%	Tissue section	SP44	Yes, but low correlation	Yes, but not an independent factor
	H-score ≥ 200	32.5%			Yes, but low correlation	Yes, but not an independent factor

GCN: gene copy number; OS: overall survival; DFS: disease-free survival; TMA: tissue microarray.

## Data Availability

The data presented in this study are available on request from the corresponding author.

## References

[1] Birchmeier C., Birchmeier W., Gherardi E., Vande Woude G.F. (2003). Met, metastasis, motility and more. Nat. Rev. Mol. Cell. Biol..

[2] Frampton G.M., Ali S.M., Rosenzweig M., Chmielecki J., Lu X., Bauer T.M., Akimov M., Bufill J.A., Lee C., Jentz D. (2015). Activation of MET via diverse exon 14 splicing alterations occurs in multiple tumor types and confers clinical sensitivity to MET inhibitors. Cancer Discov..

[3] Drilon A., Cappuzzo F., Ou S.I., Camidge D.R. (2017). Targeting MET in Lung Cancer: Will Expectations Finally Be MET?. J. Thorac. Oncol..

[4] Dagogo-Jack I., Yoda S., Lennerz J.K., Langenbucher A., Lin J.J., Rooney M.M., Prutisto-Chang K., Oh A., Adams N.A., Yeap B.Y. (2020). MET Alterations Are a Recurring and Actionable Resistance Mechanism in ALK-Positive Lung Cancer. Clin. Cancer Res..

[5] Wu Y.L., Zhang L., Kim D.W., Liu X., Lee D.H., Yang J.C., Ahn M.J., Vansteenkiste J.F., Su W.C., Felip E. (2018). Phase Ib/II Study of Capmatinib (INC280) Plus Gefitinib After Failure of Epidermal Growth Factor Receptor (EGFR) Inhibitor Therapy in Patients With EGFR-Mutated, MET Factor-Dysregulated Non-Small-Cell Lung Cancer. J. Clin. Oncol..

[6] Ou S.H., Kwak E.L., Siwak-Tapp C., Dy J., Bergethon K., Clark J.W., Camidge D.R., Solomon B.J., Maki R.G., Bang Y.J. (2011). Activity of crizotinib (PF02341066), a dual mesenchymal-epithelial transition (MET) and anaplastic lymphoma kinase (ALK) inhibitor, in a non-small cell lung cancer patient with de novo MET amplification. J. Thorac. Oncol..

[7] Spigel D.R., Edelman M.J., O’Byrne K., Paz-Ares L., Shames D.S., Yu W., Paton V.E., Mok T. (2014). Onartuzumab plus erlotinib versus erlotinib in previously treated stage IIIb or IV NSCLC: Results from the pivotal phase III randomized, multicenter, placebo-controlled MET Lung (OAM4971g) global trial. J. Clin. Oncol..

[8] Noonan S.A., Berry L., Lu X., Gao D., Baron A.E., Chesnut P., Sheren J., Aisner D.L., Merrick D., Doebele R.C. (2016). Identifying the Appropriate FISH Criteria for Defining MET Copy Number-Driven Lung Adenocarcinoma through Oncogene Overlap Analysis. J. Thorac. Oncol..

[9] Yin W., Cheng J., Tang Z., Toruner G., Hu S., Guo M., Robinson M., Medeiros L.J., Tang G. (2020). MET Amplification (MET/CEP7 Ratio ≥ 1.8) Is an Independent Poor Prognostic Marker in Patients with Treatment-naive Non-Small-cell Lung Cancer. Clin. Lung Cancer.

[10] Schildhaus H.U., Schultheis A.M., Ruschoff J., Binot E., Merkelbach-Bruse S., Fassunke J., Schulte W., Ko Y.D., Schlesinger A., Bos M. (2015). MET amplification status in therapy-naive adeno- and squamous cell carcinomas of the lung. Clin. Cancer Res..

[11] Go H., Jeon Y.K., Park H.J., Sung S.W., Seo J.W., Chung D.H. (2010). High MET gene copy number leads to shorter survival in patients with non-small cell lung cancer. J. Thorac. Oncol..

[12] Cappuzzo F., Marchetti A., Skokan M., Rossi E., Gajapathy S., Felicioni L., Del Grammastro M., Sciarrotta M.G., Buttitta F., Incarbone M. (2009). Increased MET gene copy number negatively affects survival of surgically resected non-small-cell lung cancer patients. J. Clin. Oncol..

[13] Bean J., Brennan C., Shih J.Y., Riely G., Viale A., Wang L., Chitale D., Motoi N., Szoke J., Broderick S. (2007). MET amplification occurs with or without T790M mutations in EGFR mutant lung tumors with acquired resistance to gefitinib or erlotinib. Proc. Natl. Acad. Sci. USA.

[14] Engelman J.A., Zejnullahu K., Mitsudomi T., Song Y., Hyland C., Park J.O., Lindeman N., Gale C.M., Zhao X., Christensen J. (2007). MET amplification leads to gefitinib resistance in lung cancer by activating ERBB3 signaling. Science.

[15] Lai G.G.Y., Lim T.H., Lim J., Liew P.J.R., Kwang X.L., Nahar R., Aung Z.W., Takano A., Lee Y.Y., Lau D.P.X. (2019). Clonal MET Amplification as a Determinant of Tyrosine Kinase Inhibitor Resistance in Epidermal Growth Factor Receptor-Mutant Non-Small-Cell Lung Cancer. J. Clin. Oncol..

[16] Chen H.-J., Mok T.S., Chen Z.-H., Guo A.-L., Zhang X.-C., Su J., Wu Y.-L. (2009). Clinicopathologic and molecular features of epidermal growth factor receptor T790M mutation and c-MET amplification in tyrosine kinase inhibitor-resistant Chinese non-small cell lung cancer. Pathol. Oncol. Res..

[17] Guo R., Berry L.D., Aisner D.L., Sheren J., Boyle T., Bunn P.A., Johnson B.E., Kwiatkowski D.J., Drilon A., Sholl L.M. (2019). MET IHC Is a Poor Screen for MET Amplification or MET Exon 14 Mutations in Lung Adenocarcinomas: Data from a Tri-Institutional Cohort of the Lung Cancer Mutation Consortium. J. Thorac. Oncol..

[18] Sequist L.V., Waltman B.A., Dias-Santagata D., Digumarthy S., Turke A.B., Fidias P., Bergethon K., Shaw A.T., Gettinger S., Cosper A.K. (2011). Genotypic and histological evolution of lung cancers acquiring resistance to EGFR inhibitors. Sci. Transl. Med..

[19] Yu H.A., Arcila M.E., Rekhtman N., Sima C.S., Zakowski M.F., Pao W., Kris M.G., Miller V.A., Ladanyi M., Riely G.J. (2013). Analysis of tumor specimens at the time of acquired resistance to EGFR-TKI therapy in 155 patients with EGFR-mutant lung cancers. Clin. Cancer Res..

[20] Yin W., Liu W., Guo M., Tang Z., Toruner G., Robinson M., Cheng J., Hu S., Medeiros L.J., Tangm G. (2021). Acquired MET amplification in non-small cell lung cancer is highly associated with the exposure of EGFR inhibitors and may not affect patients’ outcome. Exp. Mol. Pathol.

[21] Moosavi F., Giovannetti E., Saso L., Firuzi O. (2019). HGF/MET pathway aberrations as diagnostic, prognostic, and predictive biomarkers in human cancers. Crit. Rev. Clin. Lab. Sci..

[22] Miller C.T., Lin L., Casper A.M., Lim J., Thomas D.G., Orringer M.B., Chang A.C., Chambers A.F., Giordano T.J., Glover T.W. (2006). Genomic amplification of MET with boundaries within fragile site FRA7G and upregulation of MET pathways in esophageal adenocarcinoma. Oncogene.

[23] Tong J.H., Yeung S.F., Chan A.W., Chung L.Y., Chau S.L., Lung R.W., Tong C.Y., Chow C., Tin E.K., Yu Y.H. (2016). MET Amplification and Exon 14 Splice Site Mutation Define Unique Molecular Subgroups of Non-Small Cell Lung Carcinoma with Poor Prognosis. Clin. Cancer Res..

[24] Park S., Choi Y.L., Sung C.O., An J., Seo J., Ahn M.J., Ahn J.S., Park K., Shin Y.K., Erkin O.C. (2012). High MET copy number and MET overexpression: Poor outcome in non-small cell lung cancer patients. Histol. Histopathol..

[25] Tsuta K., Kozu Y., Mimae T., Yoshida A., Kohno T., Sekine I., Tamura T., Asamura H., Furuta K., Tsuda H. (2012). C-MET/phospho-MET protein expression and MET gene copy number in non-small cell lung carcinomas. J. Thorac. Oncol..

[26] Mignard X., Ruppert A.M., Antoine M., Vasseur J., Girard N., Mazieres J., Moro-Sibilot D., Fallet V., Rabbe N., Thivolet-Bejui F. (2018). C-MET Overexpression as a Poor Predictor of MET Amplifications or Exon 14 Mutations in Lung Sarcomatoid Carcinomas. J. Thorac. Oncol..

[27] Rivalland G., Mitchell P., Murone C., Asadi K., Morey A.L., Starmans M., Boutros P.C., Walkiewicz M., Solomon B., Wright G. (2019). Mesenchyme to epithelial transition protein expression, gene copy number and clinical outcome in a large non-small cell lung cancer surgical cohort. Transl. Lung Cancer Res..

[28] Bubendorf L., Dafni U., Schobel M., Finn S.P., Tischler V., Sejda A., Marchetti A., Thunnissen E., Verbeken E.K., Warth A. (2017). Prevalence and clinical association of MET gene overexpression and amplification in patients with NSCLC: Results from the European Thoracic Oncology Platform (ETOP) Lungscape project. Lung Cancer.

[29] Dziadziuszko R., Wynes M.W., Singh S., Asuncion B.R., Ranger-Moore J., Konopa K., Rzyman W., Szostakiewicz B., Jassem J., Hirsch F.R. (2012). Correlation between MET gene copy number by silver in situ hybridization and protein expression by immunohistochemistry in non-small cell lung cancer. J. Thorac. Oncol..

[30] Tsakonas G., Botling J., Micke P., Rivard C., LaFleur L., Mattsson J., Boyle T., Hirsch F.R., Ekman S. (2019). c-MET as a biomarker in patients with surgically resected non-small cell lung cancer. Lung Cancer.

[31] Weingertner N., Meyer N., Voegeli A.C., Guenot D., Renaud S., Massard G., Falcoz P.E., Olland A., Mennecier B., Gaub M.P. (2015). Correlation between MET protein expression and MET gene copy number in a Caucasian cohort of non-small cell lung cancers according to the new IASLC/ATS/ERS classification. Pathology.

[32] Koeppen H., Yu W., Zha J., Pandita A., Penuel E., Rangell L., Raja R., Mohan S., Patel R., Desai R. (2014). Biomarker analyses from a placebo-controlled phase II study evaluating erlotinib+/-onartuzumab in advanced non-small cell lung cancer: MET expression levels are predictive of patient benefit. Clin. Cancer Res..

[33] Pyo J.S., Kang G., Cho W.J., Choi S.B. (2016). Clinicopathological significance and concordance analysis of c-MET immunohistochemistry in non-small cell lung cancers: A meta-analysis. Pathol. Res. Pract..

[34] Patane S., Avnet S., Coltella N., Costa B., Sponza S., Olivero M., Vigna E., Naldini L., Baldini N., Ferracini R. (2006). MET overexpression turns human primary osteoblasts into osteosarcomas. Cancer Res..

[35] Wang R., Ferrell L.D., Faouzi S., Maher J.J., Bishop J.M. (2001). Activation of the Met receptor by cell attachment induces and sustains hepatocellular carcinomas in transgenic mice. J. Cell. Biol..

[36] Wu Y.L., Cheng Y., Zhou J., Lu S., Zhang Y., Zhao J., Kim D.W., Soo R.A., Kim S.W., Pan H. (2020). Tepotinib plus gefitinib in patients with EGFR-mutant non-small-cell lung cancer with MET overexpression or MET amplification and acquired resistance to previous EGFR inhibitor (INSIGHT study): An open-label, phase 1b/2, multicentre, randomised trial. Lancet Respir. Med..

[37] Casadevall D., Gimeno J., Clave S., Taus A., Pijuan L., Arumi M., Lorenzo M., Menendez S., Canadas I., Albanell J. (2015). MET expression and copy number heterogeneity in nonsquamous non-small cell lung cancer (nsNSCLC). Oncotarget.

